# Supplementary material for: Feasibility of a community-based intervention for the diagnosis and management of hypertension in two rural populations in Kenya and The Gambia: IMPLEMENT-IHCoR feasibility study protocol
Source: BMJ Open. 2026 Jun 9;16(6):e108926. doi: 10.1136/bmjopen-2025-108926 (PMC13264890; doi:10.1136/bmjopen-2025-108926)
Supplement: online supplemental file 2 [file bmjopen-16-6-s002.docx]

**Appendix 2 A-J**

## 2A – FGD topic guide – Patient (Round 1)

**BLOOD PRESSURE: PREVIOUS EXPERIENCES/ AWARENESS**

1. Have you heard about high blood pressure (hypertension) before your blood pressure was measured?

Probe: awareness of symptoms, potential complications, views about causes of hypertension

1. Before you came to [name] health centre, were you already aware of ways to manage pressure?

Probe: can you give examples? diet, physical activity, medication

1. Where did you learn about it?

Probe: any experience of someone in your family/someone you know who has lived with high blood pressure? How was it like for them? Health centre? CHPs?

**COMMUNITY SCREENING & REFERRAL**

1. How did you feel about being screened for blood pressure at home?

Probe: What did you like/dislike? Can you tell me why?

And how about it being done by the CHPs?

What did you like/dislike? Can you tell me why?

Did you experience any challenges during blood pressure measurement? How were they resolved?

1. What information were you given after your blood pressure was measured?

Probe: BP reading, referral information. How did you feel about the information you were given by the CHP? Did you have any questions for the CHP?

1. How did you arrange to get to the facility? Were there any challenges? How did you resolve these challenges?

**CLINIC PROCESS & TREATMENT**

1. Now can we talk about your experience of the clinic assessment [height etc measurements, BP, Urine, ECG ‘tested for the heart’] at [name] facility?

Probe: How did you feel about these procedures? How they were done? How they were explained to you?

1. Can you tell me how your results were explained to you?

Probe: what did the (nurse/doctor) tell you? How did you feel about this? Did you feel you understood/could ask questions?

1. What information were you given about ways to reduce your blood pressure?

Probe: What kind of things were you told about (diet, physical activity)?

How did you feel about this advice?

Do you think it is something you want to do? Can do? Why? Why not?

1. Can you tell me about your experience of this [lifestyle] advice so far?

Any challenges you have experienced? How have you resolved these challenges?

1. Are there any specific changes you have made, and if so how have you gone about it? How does it fit/not in your day? Is there anyone who helps you with these changes? How so?

**OVERALL EXPERIENCE**

1. How well do you think the blood pressure care we’ve discussed fits (or not) with people’s practices in your community?

Probe: herbal medicine, we are interested to learn beliefs about health, illness, alternative treatment/medicine in your community, spiritual beliefs?

1. Do you have any questions we haven’t covered?

**[Close and thank the participants]**

## 2B – FGD topic guide – Patient (Round 2)

Note: In Month 6, we aim to gather insights on participant application of lifestyle advice so far and BP measurement at Month 6.

**BLOOD PRESSURE: PREVIOUS EXPERIENCES/ AWARENESS**

1. Had you heard about high blood pressure (hypertension) before your blood pressure was measured?

Probe: awareness of symptoms, potential complications, views about causes of hypertension

1. Before you came to [name] health centre, were you already aware of ways to manage pressure?

Probe: can you give examples? diet, physical activity, medication

1. Where did you learn about it?

Probe: any experience of someone in your family/someone you know who has lived with high blood pressure? How was it like for them? Health centre? CHPs?

**COMMUNITY SCREENING & REFERRAL**

1. How did you feel about being screened for blood pressure at home?

Probe: What did you like/dislike? Can you tell me why?

1. And how about it being done by the CHPs?

Probe: What did you like/dislike? Can you tell me why?

1. Did you experience any challenges during blood pressure measurement? How were they resolved?
2. What information were you given after your blood pressure was measured?
3. Probe: BP reading, referral information. How did you feel about the information you were given by the CHP? Did you have any questions for the CHP?
4. How did you arrange to get to the facility? Were there any challenges? How did you resolve these challenges?

**CLINIC PROCESS & TREATMENT**

1. Now can we talk about your experience of the clinic assessment [height etc measurements, BP, Urine, ECG ‘tested for the heart’] at [name] facility?

Probe: How did you feel about these procedures? How they were done? How they were explained to you?

1. Can you tell me how your results were explained to you?

Probe: what did the (nurse/doctor) tell you? How did you feel about this? Did you feel you understood/could ask questions?

1. What information were you given about ways to reduce your blood pressure?

Probe: What kind of things were you told about (diet, physical activity)? What are some specific things you were told about that you could do? How easy has it been do this?

How did you feel about this advice?

Do you think it is something you want to do? Can do? Why? Why not?

How do you feel about it now, six months later?

1. Can you tell me about your experience of this [lifestyle] advice so far? Any challenges you have experienced? How have you resolved these challenges? Has anything changed over time since you were given the advice?
2. Are there any specific changes you have made, and if so how have you gone about it? Are there some who have not made changes, what might be helpful in future to help with changes? How does it fit/not in your day? Is there anyone who helps you with these changes? Or is involved in, or affected by these changes? How so?

**MONTH SIX BP MEASUREMENT**

1. How did you feel about the follow-up blood pressure measurement done by the CHP at home?

Probe: What did you like/dislike? Can you tell me why?

Did you experience any challenges during blood pressure measurement? How were they resolved?

1. What information were you given after your blood pressure was measured?

Probe: BP reading. How did you feel about the information you were given by the CHP? Did you have any questions for the CHP?

**OVERALL EXPERIENCE**

1. How well do you think the blood pressure care we’ve discussed fits (or not) with people’s practices in your community?
2. Probe: herbal medicine, we are interested to learn beliefs about health, illness, alternative treatment/medicine in your community, spiritual beliefs?
3. Do you have any questions we haven’t covered?

**[Close and thank the participants]**

## 2C – IDI topic guide – Patient (Round 1)

Note: In Month 1, we aim to gather insights regarding participant experience of community screening, referral, diagnosis (including clinic assessment at the facility) and treatment initiation.

**GENERAL HEALTH BACKGROUND**

1. Can you tell me about your health generally?

Probe: Is there anything that bothers you (symptoms, common ailments)?

1. Do you have any conditions that you visit the health facility or regularly take medication for?

Probe: How do you find it? Any challenges with taking medication regularly?

If yes, how do you cope with these challenges?

1. Have you heard about high blood pressure (hypertension) before you were diagnosed?

Probe: awareness of symptoms, potential complications, views about causes of hypertension, awareness of ways to manage (diet, medication).

1. Do you have any experience of someone in your family/someone you know who has lived with high blood pressure? How was it like for them?

**COMMUNITY SCREENING & REFERRAL**

1. Can you describe what happened when the CHP came to your home to take your blood pressure measurement?

Probe: Where was the blood pressure measurement done, who else was present?

1. Did you experience any challenges during blood pressure measurement? How were they resolved?
2. How did you feel about being screened for blood pressure at home?

Probe: What did you like/dislike? Can you tell me why?

1. And how about it being done by the CHPs?

Probe: What did you like/dislike? Can you tell me why?

1. How did blood pressure screening at home fit into your daily schedule?

Probe: How does it compare with when you have to go to the facility?

1. Could you tell me what happened after your blood pressure was measured?

Probe: What information were you given—BP reading, referral information. How did you feel about the information you were given by the CHP? Did you have any questions for the CHP?

1. How did you arrange to get to the facility? Were there any challenges? How did you resolve these challenges?

**CLINIC PROCESS & TREATMENT**

1. Now can we talk about what happened when you got to the facility. Can you tell me what happened?

Probe: What happened first?

- Height, weight and waist circumference measurement: What was your experience?
- Blood pressure measurement: How did it compare being done at the facility to being done at home?

Probe: How did you feel about it? How it was done? How it was explained to you?

- Urine sample: What was your experience of providing a urine sample?

Probe: How did you feel about it? How it was done? How it was explained to you?

- ECG: What was your experience of being ‘tested for the heart’?

Probe: How did you feel about where it was done? How it was done (Sticking and removing electrodes)? Needing to be undressed? How it was explained to you?

1. Can you tell me how your results were explained to you?

Probe: what did the (nurse/doctor) tell you? How did you feel about this?

1. What information were you given about ways to reduce your blood pressure?

Probe: What kind of things were you told about (diet, physical activity)? How did you feel about this advice? Do you think it is something you want to do? Can do? Why? Why not?

1. How did you feel about being prescribed medication for high blood pressure?

Probe: Feelings about taking medication regularly?

1. Can you tell me what you were told about the medication you were given?

Probes: What information were you told about the medication and how to take it? Can you tell me what this medication does in your body? How did you feel about the medication you were given?

1. Can you tell me about your experience of taking the medication so far?

Probe: time medication is taken, how do you fit in your day? How do you remember? How have you been feeling? Any challenges you have experienced? How have you resolved these challenges?

**OVERALL EXPERIENCE**

1. Thinking about your overall experience so far since the CHP first visited your home, did you feel that you could ask any questions you had?

If yes, what kind of things did you ask about? Were you satisfied with how the questions were answered?

1. How well do you think the blood pressure care we’ve discussed fits (or not) with people’s practices and beliefs about health and medicine and treatment in your community?

Probe: beliefs about health, illness, alternative treatment/medicine in your community, spiritual beliefs? And your own beliefs and values?

1. Looking ahead, can you tell me what will happen next in your blood pressure care?

Probe: (CHPs checking blood pressure and delivering medication to them). How do you feel about it?

1. Is there anyone that has supported you in coming to the health facility or managing your blood pressure?

Probe: Relationship with you? How do they support you (preparing food; medication, transport)?

1. Do you have any questions we haven’t covered?

**[Close and thank the participants]**

## 2D – IDI topic guide – Patient (Round 2)

Note: When arranging the interview, please ask the participant to bring their blood pressure medication with them to the interview. Prior to the interview, please arrange for the interviewer to have a sample of the SPC medication to show as a reference point.

Note: In Month 3-4, we aim to gather insights regarding participant experience of taking blood pressure medication and follow up (BP monitoring, adherence check, medication refill).

**GENERAL HEALTH**

1. Can you tell me about how your health is generally at the moment?  Since we last spoke, are there any new health issues that require you to visit the health facility or regularly take medication?

Probes: When was this diagnosed? How did the diagnosis happen? How is this going for you? Are you experiencing any challenges (e.g. accessing medications)? If yes, how do you cope with these challenges?

Now I would like to talk to you about your blood pressure medication:

**EXPERIENCE WITH BLOOD PRESSURE MEDICATION**

1. Can you tell me what medication you are taking for your blood pressure?

Probe: do you have it with you? can you show me? [if participant does not have it, show sample] Is there any other medication you are taking for your blood pressure?

1. Can you tell me what information you were given about your blood pressure medication?

Probe: How long you will take it for? Can you tell me about what this medication does in your body?

1. How did you feel about being prescribed medication for high blood pressure?

Probe: Feelings about taking medication regularly? How did you feel about the medication you were given?

1. Are you familiar with combination pills to treat blood pressure, or any other condition?

[if yes: can you tell me about these?]

[if no: There are some combination pills available in Kenya and other countries, which combine multiple existing blood pressure drugs into one pill. Is that something you have heard about? if yes: can you tell me about these?]

1. Since you started taking blood pressure medication, has the doctor/nurse changed your medication at all?

[IF MEDICATION HAS NOT BEEN CHANGED go to (8)]

1. Can you tell me about your experience of taking the medication so far?

Probes: how many pills do you take? how often? time medication is taken? when in your daily routine?

How do you remember? How have you been feeling since you started taking the medication?

Any challenges you have experienced? How have you resolved these challenges? Is there anyone who supports/assists you with taking your medication? In what way?

[NOW SKIP to (12)]

[IF MEDICATION HAS BEEN CHANGED go to (9)]

1. Can you tell me about how that change happened?

Probe: When did it happen, why was it changed? What information were you given? How did you feel about the change

1. Can you tell me about your experience of taking the medication so far?

Probes: how many pills do you take? how often? time medication is taken? when in your daily routine?

How do you remember? How have you been feeling since you started taking the medication?

Any challenges you have experienced? How have you resolved these challenges? Is there anyone who supports/assists you with taking your medication? In what way?

1. If your medication was changed, what was your experience of taking medication before and after the change? How did it compare?

[NOW CONTINUE to (12)]

1. Have you discussed your blood pressure medication with anyone?

Probes: Family, friends, neighbours, people you work with? What are their reactions?

1. Is there anyone else in your household with high blood pressure? If yes, what are their thoughts on your blood pressure medications?
2. How do they manage their blood pressure? Are they able to access medication? If yes, where from?

[if they are not able to access medications] How do you feel about you having this medication and them not having medication? How have you managed this?

1. What about other people in your community? How do they feel about blood pressure medication you are currently taking?

Probes: Any advice, doubts, challenges? How do people feel about this kind of medicine compared with herbal medicine?

**COMMUNITY LEVEL MONITORING & REFILL**

Now we would like to talk about follow-up visits at home from the CHP:

1. How many times has the CHP visited your home since you started taking theblood pressure medication from the facility?

[For each visit, repeat the following questions]

1. Could you tell me about your experiences when the CHP [field doctor] came to your home for the follow-up visit? [the first time/ the second time….]

Was it the same CHP who initially screened?

What did the CHP do?

- BP check (how was your BP?)
- pill check (how was the pill check?)

Did you receive a medication refill?

Probes: how/when did you receive it? was it from the same CHP? For how long (weeks?)?

What information were you given?

Probes: lifestyle advice, taking medication

1. How did you feel about the BP check, medication refill, being done by the CHP [field doctor]?
2. How did you feel about having the follow-up visit at your home? (instead of being asked to return to the health facility)

**CLINIC REFERRAL**

[if the patient was referred to come to clinic]

1. What information were you given by the CHP about coming back to the facility? How did you arrange to come to the facility?
2. Were there any challenges? How did you resolve them?
3. Can you tell me what happened at the clinic?

Probes: What information were you given? How did you feel about the clinic visit?

**OVERALL EXPERIENCE**

1. Overall, have you made any changes to your lifestyle since you started taking medication?

Probe: diet, exercise, [stress/ ‘thinking too much’ management if raised earlier ) How have you gone about it? How does it fit/not in your day?

Is there anyone who helps you with these changes? How so?

1. Do you think making changes to your lifestyle is useful to manage blood pressure?

Any challenges? If so, how have you managed these?

What information were you given about ways to reduce your blood pressure?
Probe: What kind of things were you told about (diet, physical activity)? How did you feel about this advice?

1. Thinking about your overall experience so far since the CHP first visited your home, did you feel that you could ask any questions you had?

If yes, what kind of things did you ask about? Were you satisfied with how the questions were answered?

1. How well do you think the blood pressure care we’ve discussed fits (or not) with spiritual beliefs in your community? And your own beliefs and values?
2. Looking ahead, can you tell me what will happen next in your blood pressure care?

Probe: (CHPs checking blood pressure and delivering medication to them). How do you feel about it?

1. Is there anyone that has supported you in managing your blood pressure?

Probe: Relationship with you? How do they support you (preparing food; medication, transport)?

1. Do you have any questions we haven’t covered?

**[Close and thank the participant**]

## 2E – IDI Topic Guide – Caregiver (Round 1)

Note: In Month 1, we aim to gather insights regarding participant experience of community screening, referral, diagnosis (including clinic assessment at the facility) and treatment initiation.

**GENERAL HEALTH BACKGROUND**

1. Can you tell me about the health of [patient i.e, father-in-law, mother, etc], generally?

Probe: Is there anything that bothers them (symptoms, common

ailments)?

1. Does [patient] have any conditions that they visit the health facility or regularly take medication for?

Probe: How do they find it? Any challenges with taking medication regularly?

If yes, how do they cope with these challenges? Is there anything that you do to support them with this health condition/challenge?

1. How has that experience been for you? Probe: Providing support? Is there anyone else that supports the patient? Any challenges experienced?
2. Have you (the caregiver) heard about high blood pressure (hypertension) before they (patient) were diagnosed?

Probe: awareness of symptoms, potential complications, views about causes of hypertension, awareness of ways to manage (diet, medication).

Do you know if [patient] had heard about high blood pressure (hypertension) before they were diagnosed?

Do you have any experience of someone else in your family/someone you know who has lived with high blood pressure? How was it like for them?

**COMMUNITY SCREENING & REFERRAL**

1. Can you describe what happened when the CHP came to take your (patient) blood pressure measurement at home?

Probe: Where was the blood pressure measurement done, who else was present? If not present—did your (patient) tell you anything about it? What did they say happened?

1. Were there any challenges during blood pressure measurement? How were they resolved?
2. How did you feel about (patient) being screened for blood pressure at home?

Probe: What did you like/dislike? Can you tell me why?

How about it being done by the CHPs?

Probe: What did you like/dislike? Can you tell me why?

1. How did blood pressure screening at home fit into your or (patient) daily schedule?

Probe: How does it compare with when they have go to the facility?

1. Could you tell me what happened after their blood pressure was measured?

Probe: Was any information given to you/(patient)? What information were you/(patient) given—BP reading, referral information. How did you feel about the information given by the CHP? Did you have any questions for the CHP?

1. How did you/ (patient) arrange to get to the facility? Were there any challenges? How were these challenges resolved?

**CLINIC PROCESS & TREATMENT**

1. Now, can we talk about what happened at the facility? Did you accompany your (patient) to the health facility? Probe: Can you tell me what happened? What happened first?

- Height, weight and waist circumference measurement: What did you think of how it was done?
- Blood pressure measurement: How did it compare being done at the facility to being done at home?

Probe: How it was explained? What did you think about the explanation?

- Urine sample: How was the process of (patient) providing a urine sample?

Probe: How it was explained to you?

- ECG: How was the process of (patient) being ‘tested for the heart’?

Probe: How did you feel about where it was done? How it was done (Sticking and removing electrodes)? (Patient)Needing to be undressed? How it was explained?

1. Can you tell me about how (patient) results were explained?

Probe: what did the (nurse/doctor) say? How did you feel about this?

1. What information was given about ways to reduce blood pressure?

Probe: What kind of things were you/(the patient) told about (diet, physical activity)? How did you feel about this advice? Do you think it is something (patient) wants to do? Can do? Why? Why not?

1. How did you feel about (patient) being prescribed medication for high blood pressure?

Probe: Feelings about (patient) taking medication regularly?

1. Can you tell me what you/ (patient) were told about the medication they were given?

Probes: What information was given about the medication and how to take it? Can you tell me what this medication does? How did you feel about the medication (patient) was given?

1. Can you tell me about (patient) experience of taking the medication so far?

Probe: time medication is taken, how does it fit in their day? How do they remember? How have they been feeling? Any challenges they (patient) have experienced? How have these been resolved?

1. Is there anything that you have needed to do to support the (patient) in taking their medication? Probe: specific things done to support, reminding them to take their medication, how does it fit in their day? Any challenges you have experienced? How have these been resolved?
2. Can you tell me about any other things you have needed to do to support the (patient) with blood pressure management?

Probe: cooking foods advised, exercise, diet

**OVERALL EXPERIENCE**

1. Thinking about the overall experience so far since the CHP first visited (patient), did you feel that you could ask any questions you had?

If yes, what kind of things did you ask about? Were you satisfied with how the questions were answered?

1. How well do you think the blood pressure care we’ve discussed fits (or not) with people’s practices and beliefs about health and medicine and treatment in your community?

Probe: beliefs about health, illness, alternative treatment/medicine in your community, spiritual beliefs? And your own beliefs and values?

1. Looking ahead, can you tell me what will happen next in (patients') blood pressure care?

Probe: (CHPs checking blood pressure and delivering medication to them). How do you feel about it?

1. Do you have any questions we haven’t covered?

**[Close and thank the participant]**

**IDI Topic guide for Caregiver/Round 2**

Note: When arranging the interview, please arrange for the interviewer to have a sample of the SPC medication to show as a reference point.

Note: In Month 3-4, we aim to gather insights regarding participant experience of taking blood pressure medication and follow up (BP monitoring, adherence check, medication refill).

**GENERAL HEALTH BACKGROUND**

1. Can you tell me about how your (patient) health is generally at the moment?  Since we last spoke, are there any new health issues that require him/her to visit the health facility or regularly take medication?

Probes: When was this diagnosed? How did the diagnosis happen? How is this going for him/her? Are they (patient) experiencing any challenges (e.g. accessing medications)? If yes,  how do they cope with these challenges?

1. How has that experience been for you? Probe: Providing support? Any challenges experienced?
2. Is there anyone else that supports the patient, regularly or occasionally? Probe: support with health? Support with other things in daily life? What kinds of tasks? Any challenges experienced?

**EXPERIENCE WITH BLOOD PRESSURE MEDICATION**

1. Can you tell me what medication your (patient) is taking for blood pressure?

[show sample] Is there any other medication your (patient) is taking for their blood pressure?

1. Can you tell me what information your (patient) was given about their blood pressure medication?

Probe: How long they will take it for? Can you tell me about what this medication does in their body?

1. How did you feel about your (patient) being prescribed medication for high blood pressure?

Probe: Feelings about the patient taking medication regularly? How did you feel about the medication your (patient) was given

1. Are you familiar with combination pills to treat blood pressure, or any other condition?

[if yes: can you tell me about these?]

[if no: There are some combination pills available in Kenya and other countries, which combine multiple existing blood pressure drugs into one pill. Is that something you have heard about? if yes: can you tell me about these?

1. Since your ([patient) started taking blood pressure medication, has the doctor/nurse changed their medication at all?

[IF MEDICATION HAS BEEN CHANGED go to (9)]

[IF MEDICATION HAS NOT BEEN CHANGED go to (10)]

1. Can you tell me about how that change happened?

Probe: When did it happen, why was it changed? What information was your (patient) given? How did they feel about the change?

1. Can you tell me about your (patient) experience of taking the medication so far?

Probes: how many pills do they take? how often? time medication is taken? when in their daily routine? How do they remember?

How has your (patient) been feeling since they started taking the medication?

Any challenges you have noted since they started using that medication? Challenges your (patient) has complained about? How have these challenges been resolved?

[NOW CONTINUE to (10)]

1. Is there anyone else in your household with high blood pressure? If yes, what are their thoughts on your (patient’s) blood pressure medications?

How do they manage their blood pressure? Are they able to access medication? If yes, where from?

[if they are not able to access medications] How do you feel about your (patient) having this medication and them not having medication? How have you/ they managed this?

1. What about other people in your community? How do they feel about blood pressure medication your (patient) is currently taking?

Probes: Any advice, doubts, challenges?

How do people feel about this kind of medicine compared with herbal medicine?

**COMMUNITY LEVEL MONITORING & REFILL**

Now we would like to talk about follow-up visits at home from the CHP:

1. How many times has the CHP visited your home since your (patient) started taking the blood pressure medication from the facility?

[For each visit, repeat the following questions:]

1. Could you tell me about your experiences when the CHP [field doctor] came to your (patient’s) home for the follow-up visit? [the first time/ the second time….] (if you were there/heard about it…)

Was it the same CHP who initially screened?

What did the CHP do?

BP check (how was your (patient) BP?)

pill check (how was the pill check?)

did your ([patient] receive a medication refill?

Probes: how/when did you receive it? was it from the same CHP? For how long (weeks)?

What information were you/ your (patient) given?

Probes: lifestyle advice, taking medication

1. How did you feel about the BP check, medication refill, being done by the CHP [field doctor]?
2. How did you feel about having the follow-up visit at your (patient’s) home? (instead of your (patient) being asked to return to the health facility)

**CLINIC REFERRAL**

[if the patient was referred to come to clinic]

1. What information were you/ your (patient) given by the CHP about coming back to the facility? How did you/ your (patient) arrange to come to the facility? Did you/someone else accompany them?

Were there any challenges? How did you/ your (patient) resolve them?

1. Can you tell me what happened at the clinic?

Probes: What information were you/ your (patient) given? How did you feel about the clinic visit?

**OVERALL EXPERIENCE**

1. Overall, has your (patient) made any changes to their lifestyle since they started taking medication? Probe: diet, exercise, [stress/ ‘thinking too much’ management if raised earlier).

Do you think making changes to their lifestyle is useful to manage blood pressure?

1. Thinking about the overall experience so far since the CHP first visited (patient), did you feel that you could ask any questions you had?

If yes, what kind of things did you ask about? Were you satisfied with how the questions were answered?

1. How well do you think the blood pressure care we’ve discussed fits (or not) with spiritual beliefs in your community? And your own beliefs and values?
2. Looking ahead, can you tell me what will happen next in your (patient) blood pressure care?

Probe: (CHPs checking blood pressure and delivering medication to them). How do you feel about it?

1. Do you have any questions we haven’t covered?

**[Close and thank the participant]**

## 2F – IDI topic guide – Caregiver (Round 2)

Note: When arranging the interview, please arrange for the interviewer to have a sample of the SPC medication to show as a reference point.

Note: In Month 3-4, we aim to gather insights regarding participant experience of taking blood pressure medication and follow up (BP monitoring, adherence check, medication refill).

**GENERAL HEALTH BACKGROUND**

1. Can you tell me about how your (patient) health is generally at the moment?  Since we last spoke, are there any new health issues that require him/her to visit the health facility or regularly take medication?

Probes: When was this diagnosed? How did the diagnosis happen? How is this going for him/her? Are they (patient) experiencing any challenges (e.g. accessing medications)? If yes, how do they cope with these challenges?

1. How has that experience been for you?

Probe: Providing support? Any challenges experienced?

1. Is there anyone else that supports the patient, regularly or occasionally?

Probe: support with health? Support with other things in daily life? What kinds of tasks? Any challenges experienced?

**EXPERIENCE WITH BLOOD PRESSURE MEDICATION**

1. Can you tell me what medication your (patient) is taking for blood pressure?

[show sample] Is there any other medication your (patient) is taking for their blood pressure?

1. Can you tell me what information your (patient) was given about their blood pressure medication?

Probe: How long they will take it for?

Can you tell me about what this medication does in their body?

1. How did you feel about your (patient) being prescribed medication for high blood pressure?

Probe: Feelings about the patient taking medication regularly?

How did you feel about the medication your (patient) was given

1. Are you familiar with combination pills to treat blood pressure, or any other condition?

[if yes: can you tell me about these?]

[if no: There are some combination pills available in Kenya and other countries, which combine multiple existing blood pressure drugs into one pill. Is that something you have heard about? if yes: can you tell me about these?]

1. Since your ([patient) started taking blood pressure medication, has the doctor/nurse changed their medication at all?

[IF MEDICATION HAS BEEN CHANGED go to (9]

[IF MEDICATION HAS NOT BEEN CHANGED go to (10)]

1. Can you tell me about how that change happened?

Probe: When did it happen, why was it changed? What information was your (patient) given? How did they feel about the change?

1. Can you tell me about your (patient) experience of taking the medication so far?

Probes: how many pills do they take? how often? time medication is taken? when in their daily routine? How do they remember?

How has your (patient) been feeling since they started taking the medication?

Any challenges you have noted since they started using that medication? Challenges your (patient) has complained about? How have these challenges been resolved?

[NOW CONTINUE to (10)]

1. Is there anyone else in your household with high blood pressure? If yes, what are their thoughts on your (patient’s) blood pressure medications?

How do they manage their blood pressure? Are they able to access medication? If yes, where from?

[if they are not able to access medications] How do you feel about your (patient) having this medication and them not having medication? How have you/ they managed this?

1. What about other people in your community? How do they feel about blood pressure medication your (patient) is currently taking?

Probes: Any advice, doubts, challenges?

How do people feel about this kind of medicine compared with herbal medicine?

**COMMUNITY LEVEL MONITORING & REFILL**

Now we would like to talk about follow-up visits at home from the CHP:

1. How many times has the CHP visited your home since your (patient) started taking the blood pressure medication from the facility?

[For each visit, repeat the following questions:]

1. Could you tell me about your experiences when the CHP [field doctor] came to your (patient’s) home for the follow-up visit? [the first time/ the second time….] (if you were there/heard about it…)

Was it the same CHP who initially screened?

What did the CHP do?

BP check (how was your (patient) BP?)

Pill check (how was the pill check?)

Did your ([patient] receive a medication refill?

Probes: how/when did you receive it? was it from the same CHP? For how long (weeks?)?

What information were you/ your (patient) given?

Probes: lifestyle advice, taking medication

1. How did you feel about the BP check, medication refill, being done by the CHP [field doctor]?
2. How did you feel about having the follow-up visit at your (patient’s) home? (instead of your (patient) being asked to return to the health facility)

**CLINIC REFERRAL**

[if the patient was referred to come to clinic]

1. What information were you/ your (patient) given by the CHP about coming back to the facility? How did you/ your (patient) arrange to come to the facility? Did you/someone else accompany them?

Were there any challenges? How did you/ your (patient) resolve them?

1. Can you tell me what happened at the clinic?

Probes: What information were you/ your (patient) given? How did you feel about the clinic visit?

**OVERALL EXPERIENCE**

1. Overall, has your (patient) made any changes to their lifestyle since they started taking medication? Probe: diet, exercise, [stress/ ‘thinking too much’ management if raised earlier)

Do you think making changes to their lifestyle is useful to manage blood pressure?

1. Thinking about the overall experience so far since the CHP first visited (patient), did you feel that you could ask any questions you had?

If yes, what kind of things did you ask about? Were you satisfied with how the questions were answered?

1. How well do you think the blood pressure care we’ve discussed fits (or not) with spiritual beliefs in your community? And your own beliefs and values?
2. Looking ahead, can you tell me what will happen next in your (patient) blood pressure care?

Probe: (CHPs checking blood pressure and delivering medication to them). How do you feel about it?

1. Do you have any questions we haven’t covered?

**[Close and thank the participant**

## 2G – IDI topic guide – HCW/CHN (Round 1)

**ROLE AND TRAINING**

1. To start with, can you tell me about your role here at [health facility name]?

Probe: How long have you been working in this role? What are your main responsibilities?

1. What is your role/involvement in the management of patients with hypertension in your normal work here at [health facility name]?

Probe: What aspects of care/tasks are you normally responsible for in hypertension and CVD treatment/management?

1. Could you explain what your roles are in the hypertension project?

Probe: What tasks are you responsible for? Do you share this [overall role/specific task] with anyone? if yes, how did you plan this? (planned together, delegated?)

Have you or your colleagues had to make any adjustments to your normal roles to be able to do the tasks in this hypertension project

1. Could you tell me about your experience during training [recent training] for the hypertension study?

How did it fit in with your other tasks that you needed to do [in your role as X] at the facility? Did you have to make some adjustments to be able to attend the training? (Please describe these…)

How did you find the training materials?

Probe: knowledge questions, role plays and group activities, is there anything you think would have been useful to include in the training that wasn’t covered?)

Do you have any feedback on how the training was organized (location, length, timing, who was leading it, who was attending it?)?

1. Overall, how well prepared did you feel to conduct the hypertension study activities?

How confident do you feel in implementing these activities? (If not confident, what do you think would support you in becoming more confident)

**COMMUNITY SCREENING AND REFERRAL**

1. How do you feel about Community Health Promoters/Voluntary Health Workers (CHPs/VHWs) screening for hypertension in people’s homes; and referring patients to you?

Probes: confidence in how they will do this? Patients following up referrals? Share any experiences/ challenges that you have observed.

Have there been any adjustments made to the CHP/VHW screening/referral process since they started? (probe: examples, adding BP to form)

Does the hypertension screening/referral affect CHPs/VHWs' support for any other patients/ outreach projects?

Then, can we talk about the process that happens when patients come to the facility?

**PROJECT COMPONENTS (NEW/DIFFERENT PRACTICES)**

1. How do you find the project BP procedure?

How does it compare to how BP measurements are normally done in the facility?

Probes: different person’s responsibility, different machine, what do you think about the quality of the BP reading from the project BP machine?

Have you needed any support from colleagues/study team in doing the project BP measure? In what ways

Does the project BP procedure impact time/space/facilities for other patients/services? (probe: examples, bed, adjustments/workarounds)

1. How did you find the urine dipstick process?

How does it compare to how urine tests are normally done in the facility?

Probes: different person conducting the test? (new skill for this person?), different setting/room?

Does the urine test procedure impact time/space/facilities for other patients/services?

Probes: toilet for patient to use, sink/surface for test, dustbin?

Have you needed any support from colleagues/study team in doing the urine dipstick test? [if yes] In what way

1. How do you find the ECG procedure? Can you tell me about how it has been going?

Have there been any challenges/ aspects that have been more or less straightforward?

Probes: explaining to the patient, placing the electrodes, reading the result, and explaining the result to the patient? for particular age/gender/physical characteristics?

[if yes] Are there any adjustments you have made to work around challenges? (example?)

Have you needed any support from colleagues/study team in doing the ECG procedure? In what way

1. How do you find the treatment decision process in the hypertension project? [if relevant to role]

How does it compare with your normal decision process for starting treatment for other hypertensive patients?

Probe: is the categorization same/different? How do you feel about this?

How do you find the treatment decision pop up?

Probe: is it useful for you? Do you agree with it? are there any examples of patients who you would have made different decision about?

Have you needed any support from colleagues/study team in the treatment decision process? In what way

1. How do you find the lifestyle advice process?

Probe: Are there any challenges for particular topics or patient characteristics?

How does it compare to the normal process at [facility name] for giving lifestyle advice?

Probe: does it cover the same things/detail? Is it done by the same person? How do you feel about this?

Have you needed any support from colleagues/study team in the lifestyle advice process? In what way

**PRESCRIBING AND DISPENSING**

1. How do you feel about prescribing the SPC (single pill combination) medication in the hypertension project?

How does it compare to usual prescribing for hypertension at [facility name]?

Probe: which medication, combination or separate molecule, length of time prescribed for, medication availability, record keeping, tasks for patient?)

Have there been any adjustments made to the prescribing/dispensing process since the project started? (probe: examples, who does each task, where meds are stored)

Have you needed any support from colleagues/study team in the prescribing/dispensing process? In what way

1. How do you feel about the CHP/VHWs monitoring patients and giving medication refills?
2. Can you explain briefly how this compares to usual BP monitoring and follow-up at [facility name]
3. Finally, thinking about all the parts of the hypertension project together: How effective do you think it is likely to be in:

- Identifying who needs treatment to control their BP. (why/why not?)
- Supporting those people to control their BP? (why/why not?)

1. How well do you think the hypertension project activities fit (or not) with people’s practices and beliefs about health, medicine and treatment in the community?

Probe: beliefs about health, illness, alternative treatment/medicine, spiritual beliefs?

1. Do you have any reflections or feedback about the project and the way it is being conducted here at [health facility] that we haven’t discussed?
2. Do you have any questions we haven’t covered?

**[Close and thank the participant].**

## 2H – IDI topic guide – HCW/CHN (Round 2)

Note: In Month 3-4, we aim to gather insights regarding participant experience of providing blood pressure care for study participants (BP monitoring, medication refill, and adjustment, information provided to patients).

**ROLE**

1. To start with, can you tell me about your experience so far in providing care to hypertension study patients after they were prescribed medication?

Probe: What tasks are you responsible for? Do you share this [overall role/specific task] with anyone? if yes, how did you plan this? (planned together, delegated?)

Have you or your colleagues made any adjustments to your normal roles to be able to do the tasks in this hypertension project?

Can you tell me more about these adjustments?

**MONITORING REFERRAL TO FACILITY AND TREATMENT**

1. How do you feel about the CHP/VHWs monitoring patients, checking adherence and giving medication refills (Can you explain briefly how this compares to usual BP monitoring and follow-up at [facility name])

Probe each: monitoring patients, checking adherence, giving medication refills

1. What has been your experience of receiving patients referred to you from the community?

How do you identify the study participants referred to the facility by the CHP after follow-up visits? (specific days for referral, prior communication by CHP) (How well has this worked?

What are some of the reasons patients have been referred to the clinic? If study-related - Probe: [side effects, adherent and not controlled, and others, give examples]; If non-study related, provide examples

Have there been incidents when the participants came for a facility visit and were not able to be seen? If yes, could you tell me a bit more about that? Did you sometimes have participants referred to you, who you did not expect to be referred? If yes, how did you handle this?

1. Can you tell me what happens when you see/attend to the referred study participants?

Probe: Review community BP and adherence results, repeat BP at the facility

If Repeat BP: How does the community and facility BPs compare? Which BP measurements do you consider in making the treatment decision?

1. How do you find the treatment decision process for participants who have been referred to the facility in the hypertension project?

How does it compare with your normal decision process for providing follow-up treatment?

Probe: Is the threshold for treatment adjustment the same or different, and how do you feel about this?

What do you consider is important when making treatment decisions? Do you focus on the current BP measure, the trend/pattern of BP over time, other factors?

How do you find the treatment decision pop up?

Probe: is it useful for you? Do you agree with it? are there any examples of patients who you would have made different decision about?

Have you needed any support from colleagues/study team in the treatment decision process? In what way

Have you experienced any challenges? How were these resolved?

1. Have there been any adjustments to prescribing or dispensing processes of study medication in the hypertension project since we last spoke? (probe: examples, who does each task, where meds are stored, when patients receive study medication)

How does it compare to usual prescribing for hypertension at [facility name]?

Probe: which medication, combination or separate molecule, length of time prescribed for, medication availability, record keeping, tasks for patient?

Have you needed any support from colleagues/study team in the prescribing/dispensing process? In what way

Have you experienced any challenges in prescribing and dispensing study medication? How were these resolved?

1. What information do you give to the referred study participants after prescribing treatment?

Probe: [Lifestyle advice, side effects, adherence, specific time of taking medication, how frequent, chronic nature of hypertension?Have you found that patients have been reporting any challenges with taking medication?

Have any side effects been reported? what impact have these had? How have these been managed? Do you feel well prepared to manage these? Any areas where more training/support for staff is needed?

1. How do you think management of treatment would be affected if separate pills were being used instead of the SPC?

For you and colleagues at [facility name]? (prescribing, storing, dispensing medications? More or less easy to manage)

For patients? Do you think they would take the medication in the same way? More or less easy to manage?

1. Finally, thinking about all the parts of the hypertension project together: How effective do you think it has been in:

Supporting those people to control their BP? (why/why not?)

How well do you think the blood pressure care we’ve discussed fits (or not) with spiritual beliefs in your community? And your own beliefs and values?

1. Do you have any reflections or feedback about the project and the way it is being conducted here at [health facility] that we haven’t discussed?
2. With the study coming to an end in a few months:

How do you think you will plan for the study participants to continue receiving the study drugs for the next six months?

Probes: Are there any challenges you anticipate in continuing to dispense the study medication for the next six months? Can you tell me more about these? How might these challenges be solved?

What parts of the study do you think will continue beyond the study period?

Probe for hypertension screening by CHPs? Use of SPC? Adherence check by CHPs? Medication refill by CHPs?

Can you tell me more about why you think the aspects you have mentioned might continue? What do you think will enable them to continue?

If this project were extended to other facilities, what recommendations would you have?

1. Do you have any questions we haven’t covered?

**[Close and thank the participant].**

## 2I – IDI topic guide – CHP/VHW (Round 1)

Note: In Month 1, we aim to gather insights regarding CHP/VHW experience in attending IMPLEMENT-CVD training, locating study target participants within their homes and engaging them in the project. This includes challenges encountered, strategies employed to locate them, experiences in making initial contact with participants, conducting screening, measuring blood pressure, and referring them to health facilities. Additionally, we want to determine if CHPs/VHW conducted any follow-up with the referred participants to ascertain whether they visited the facility, and if not, to understand their reasons for not doing so. Across all of these tasks we want to learn about any adaptations or workarounds they used to address challenges, and their views on how different groups of patients (age, gender, comorbidity) responded.

**ROLE & TRAINING**

1. In your previous experience as a CHP/VHW, what kind of health issues have you supported?

Probe: immunization, polio, WASH, TB, HIV/AIDs, who they supported –mothers, babies, men, women, children

What were your specific roles/tasks?

How much time did you spend on these tasks (per week or month)?

1. Can you share any past experiences supporting individuals with hypertension? What were your primary responsibilities as a CHP/VHW?

Probes for:

Providing health advice: What type of advice, where?

Referring patients: for what reasons and to what facility levels

Screening patients

Providing /delivering treatment-when and where?

How much time (per week) did you spend on tasks such as health advice, treatment, and referring patients?

1. Could you tell me about your experience during training [recent training] for the hypertension study?

How did attending the training fit in with your other tasks that you needed to do in your role as a CHP, or your other responsibilities?

Probe: Did you have to make some adjustments to be able to attend the training? [if yes] please describe these

How did you find the training?

Probe: knowledge questions, role plays, group activities, paper material?

Anything you would suggest to do differently?

Do you have any feedback on how the training was organized?

Probe: location, length, timing, who was leading it, who was attending?)

1. Overall, how prepared did you feel to conduct the hypertension study activities?

How confident do you feel in implementing these activities?

Probe: [if less well prepared/ confident] what do you think would support you in becoming more prepared/confident?

**COMMUNITY SCREENING & REFERRAL**

1. How did you initially trace the participants assigned to you?
2. Were some easier or more difficult to locate than others?

Probe for who- men/women, older/younger, type of work

Did you experience any challenges? (examples?) How did you deal with these challenges?

Probe for examples - distance, rains, participant attitude, relocation, participant unavailability

1. What was your experience of introducing the hypertension project to the people you traced?

Probe: how did people respond? Did any people respond differently to others? People involved in other health initiatives, men/women, older/younger, people

How was the consenting process?

Probe for any challenges, the signing part

1. How did you feel about screening for blood pressure at people’s homes?

Probe: Can you explain why you felt that way? How did it go?

Can you explain the steps you followed when measuring participants' blood pressure?

Were there any adjustments you made during the procedure?

Probe: where did the screening happen? Did you encounter any difficulties, (such as equipment issues, participant concerns, or environmental challenges)? How did you handle them?

How do you think the process was like for the participants?

1. How did you feel about the referral process ?

Probe: Can you explain why you felt that way? How did it go?

Can you explain the steps you followed when referring participants?

Were there any adjustments you made when referring participants?

Were there any challenges faced? [if yes] How did you resolve them?

Probe: Did you use any strategies to encourage patients to go for the referral? Can you tell me about the strategies?

Did you have any participants who refused or were reluctant to follow through the referrals? (what were their reasons)

How do you think this referral process was for the participants?

1. How do you feel about monitoring patients and giving medication refills?

Can you explain why you feel that way? Is there anything you are concerned about?

Can you explain briefly how this compares to usual BP monitoring and follow-up for patients in your community?

Now, thinking about all the parts of the hypertension project together:

1. Based on your experience so far, how well do you think the hypertension project will:

Identify who needs treatment to control their BP? (why/why not?)

Support people with hypertension to control their BP? (why/why not?)

1. How has your experience working as a pair in the hypertension project been so far?

Probe: What worked well? What worked less well?

How do you think working together was for other pairs?

Probe: do you think it was similar or different for everyone?

1. How have you found that working on the hypertension project fits in (or not) with your other responsibilities?

Probe: responsibilities as a CHP/VHW, in the rest of your life

Are there any things that you had to change or give up to conduct hypertension project activities?

Probe: daily responsibilities, work-related, personal/family

1. How well do you think the hypertension project activities fit (or not) with people’s practices and beliefs about health and medicine and treatment in your community?

Probe: beliefs about health, illness, alternative treatment/medicine in your community, spiritual beliefs? And your own beliefs and values?

1. Do you have any reflections or feedback about the project and the way it is being conducted here at [health facility] that we haven’t discussed?
2. Do you have any questions we haven’t covered?

**[Close and thank the participant]**

## 2J – IDI topic guide – CHP/VHW (Round 2)

Note: In Month 3-4, we aim to gather insights regarding your experience of providing follow up care for participants on SPC at home [BP monitoring, adherence check, medication refill, referral to health facilities, health information). This also includes challenges encountered and strategies employed to resolve the challenges.

**ROLE AND TRAINING**

1. Since the study began [hypertension project], are there any new health issues you have been supporting other than this hypertension study?

Probe: immunization, polio, WASH, TB, HIV/AIDs, who they supported –mothers, babies, men, women, children)

What were your specific roles/tasks?

How much time did you spend on these tasks (per week or month)?

How much time have you been spending on the hypertension study roles/tasks?

1. How have you found that working on the hypertension project fits in (or not) with your other responsibilities?

Probe: responsibilities as a CHP/VHW, in the rest of your life

Are there any things that you have had to change or give up conducting hypertension project activities?

Probe: daily responsibilities, work-related, personal/family

**Community follow-up, medication refill and referral**

1. Could you tell me about how your experience has been finding the participants assigned to you during follow up visits?

Were some easier or more difficult to find than others?

Probe for who - men/women, older/younger, type of work

Did you experience any challenges? (examples?) How did you deal with these challenges?

Probe for: examples - distance, rains, participant attitude, relocation, participant unavailability

1. What has been your experience of doing follow up visits to participants assigned to you?

How did they (participants/household members) respond?

Were there any challenges experienced? How did you resolve the challenges?

1. How do you feel about doing follow up BP measurements in the community?

Where were the BP measurements done? (at home or other places like persons place of work etc.)

Were there any adjustments you made during the procedure?

How do you think the process was like for the participants?

What do you think about the BP measurements you have been taking?

Have you observed that they are changing for some people more than others? Have you noticed any patterns? (going up, going down, staying the same)

1. What has been your experience doing the adherence check?

Could you describe to me the steps you follow when doing the adherence check?

How easy or difficult has it been to do the adherence check?

Were there any challenges encountered? How were they resolved?

1. How do you feel about doing the adherence check?

Can you tell me why you feel that way?

How do you think the process was like for the participants?

1. What do you think about how adherence is going in general?

How people are managing their medications and reasons why they might/might not be taking it?

Do you think the patients might take the pills the same way if they were taking separate pills rather than SPC?

Have people shared any opinions about the medication with you?

1. What has been your experience of giving medication refills to the participants?

Please describe what you usually do to ensure the patient gets their medication? Probe for various strategies of delivering the medication to the participants

Is there any information you gave to the participants?

Probe: What information? Do they ask any questions or concerns? What kind of questions/concerns?

How easy or difficult has it been to do the patients medication refill?

Did you experience any challenges? How were they resolved?

How did you feel about the several trips you made to the participants’ homes? Probe for the several trips made to participants home to do BP measurements, return to the facility to pick medication refill and return to the community to take the study medication to participants; Do you have any ideas on how these trips could be made fewer?

1. How do you feel about giving medication refills to the participants?

Can you tell me why you feel that way?

Have you found that patients have been reporting any challenges with taking medication?

Probe for: understanding how to take medication, side effects

Have any side effects been reported? What impact do you think these have had?

1. For patients that you have had to refer, what has your experience been like?

Probe for issuing a referral form, reasons for referral

Were there some situations where you needed support to decide whether to refer a participant to the facility? (If yes, who provided the support (-CHA, Clinician, study team?) Could you tell me more about these instances?

How difficult or easy has it been for the participants to get to the facility following referral?

Probe: Did you use any strategies to encourage patients to go for the referral? Can you tell me about the strategies?

Did you have any participants who refused or were reluctant to follow through the referrals? (what were their reasons)

Were there any other challenges you experienced? How were they resolved?

1. How do you feel about the referral process?

Can you explain why you felt that way? How did it go?

How do you think this referral process was for the participants?

Now, thinking about all the parts of the hypertension project together:

1. Based on your experience so far, how well do you think the hypertension project has supported people with hypertension to control their BP? (why/why not?)
2. How has your experience working as a pair in the hypertension project been so far?

Probe: What worked well? What worked less well?

How do you think working together was for other pairs?

Probe: do you think it was similar or different for everyone?

1. How well do you think the blood pressure care we’ve discussed fits (or not) with spiritual beliefs in your community?And your own beliefs and values?
2. Do you have any reflections or feedback about the project and the way it is being conducted here at [health facility] that we haven’t discussed?
3. When this study comes to an end in the next few months, which aspects of hypertension care do you think you might continue to be involved in?

Probe: screening for hypertension, Adherence check, medication refill, referral of hypertensive patients to the facility?

Can you tell me a little more about why you think you will be able to continue these aspects?

1. Do you have any questions we haven’t covered?

**[Close and thank the participant].**
