# Supplementary material for: Feasibility of a community-based intervention for the diagnosis and management of hypertension in two rural populations in Kenya and The Gambia: IMPLEMENT-IHCoR feasibility study protocol
Source: BMJ Open. 2026 Jun 9;16(6):e108926. doi: 10.1136/bmjopen-2025-108926 (PMC13264890; doi:10.1136/bmjopen-2025-108926)
Supplement: Supplementary file 3 [file bmjopen-2025-108926.reviewer_comments.pdf]

PEER REVIEW HISTORY

BMJ Open publishes all reviews undertaken for accepted manuscripts. Reviewers are asked to complete a checklist review form and are provided with free text boxes to elaborate on their assessment. These free text comments are reproduced below.

ARTICLE DETAILS

Title (Provisional)

Feasibility of a community-based intervention for the diagnosis and management of hypertension in two rural populations in Kenya and The Gambia: IMPLEMENT-IHCoR Feasibility Study Protocol

Authors

Hassan, Syreen; Kagwanja, Nancy; Diallo, Brahima; Willis, Ruth; Hine, Jasmine; Brazeal, Aurelia; Obure, Vallery; Kalu, Catherine; Mwagwabi, Nyiro Clement; Shah, Anoop SV; Mumba, Noni; Oyando, Robinson; Perkins, Alexander; Nolte, Ellen; Tsofa, Benjamin; Barasa, Edwine; Perel, Pablo; Jobe, Modou; Etyang, Anthony; Murphy, Adrianna

---

VERSION 1 - REVIEW

**Reviewer**	**1**
**Name**	**Wright, David**
**Affiliation**	**University of Leicester, School of Healthcare**
**Date**	**31-Dec-2025**
**COI**	**None**

What is presented, is well presented and clear. The structure is appropriate.

My main concerns for this would be a sample size of 500 for a feasibility study - this is far more than is required for the stated aims and objectives, It also provides far more precision in the estimates than those stated as being acceptable. From my perspective there is an ethical issue with exposing a service to more people than necessary if it has not be shown to be safe or effective. A better rationale for a sample size of 500 is required, as the 'usual' number for feasibility studies s less than 100.

Given that this is a feasibility study I am unsure as to the justification of the inclusion of policy makers and those not directly involved with the research in the qualitative element. Rationale for their inclusion would be helpful as the results cannot be used to influence policy at this stage.

The planned topic guide for the qualitative work would enhance the protocol paper.

As a protocol paper, it provides an opportunity to provide a summary of the fidelity framework that will be used to check that the service is being delivered as per expectations and standard. A bullet point list would be helpful outlining fidelity expectations.

The set up or 'enactment' phase requires consideration with respect to dose, reach and fidelity. If the health care workers don't attend training when invited, do attend but don't engage with the training, training content is inadequate or delivery is poor then feasibility is diminished from the outset, The planned consideration of this element in the protocol would be helpful.

It is also usual, within a feasibility study protocol, to present the progression criteria that would be used to enable this to transition to a definitive trial.

Finally, as a health intervention, there is no consideration of safety i.e. are the right decisions being made, are patients being unexpectedly harmed by the intervention. Some description of the monitoring for safety would be helpful. It can be part of the fidelity assessment.

VERSION 1 - AUTHOR RESPONSE

Reviewer: 1
Dr. David Wright, University of Leicester comments to the Author:

- **What is presented, is well presented and clear. The structure is appropriate.**

  My main concerns for this would be a sample size of 500 for a feasibility study - this is far more than is required for the stated aims and objectives, It also provides far more precision in the estimates than those stated as being acceptable. From my perspective there is an ethical issue with exposing a service to more people than necessary if it has not be shown to be safe or effective. A better rationale for a sample size of 500 is required, as the 'usual' number for feasibility studies s less than 100.

Response: We thank the reviewer for the opportunity to clarify this issue. The figure of 500 participants refers to the number of individuals to be screened, not the number exposed to treatment. As explained in the methods section of the manuscript, based on prior data, we anticipate that approximately 45% of those screened will be referred for further clinical assessment. Of these, we expect around 25% (equivalent to approximately 10% of the total screened sample) to meet eligibility criteria and be prescribed SPC treatment. Therefore, from 500 individuals screened (250 per country), we anticipate that approximately 50 participants in total will initiate treatment.

The primary feasibility objectives relate to assessing identification, referral pathways, acceptability, treatment initiation, adherence, and follow-up processes. To meaningfully evaluate these implementation processes across two country contexts, an adequate

number of participants progressing through each stage of the pathway is required to obtain adequate information on feasibility/process outcomes and their precision. A substantially smaller screening sample (e.g., 100 individuals) would be unlikely to yield sufficient numbers initiating treatment to assess feasibility endpoints.

- **Given that this is a feasibility study I am unsure as to the justification of the inclusion of policy makers and those not directly involved with the research in the qualitative element. Rationale for their inclusion would be helpful as the results cannot be used to influence policy at this stage.**

Response: Thank you for giving us the opportunity to respond to this point. In our view, the broader policy-making context, and in particular the sustainability of the intervention within this context, is a crucial component of feasibility. We are aware that many implementation frameworks (RE-AIM; CFIR) have been revised in recent years to better account for this; yet, many interventions are evaluated in isolation, failing to account for the broader policy context. The policymakers included in our feasibility study interviews would not be completely unfamiliar with the intervention. These are policymakers that were engaged in earlier health system assessments and co-production. We have clarified this point in our revision.

- **The planned topic guide for the qualitative work would enhance the protocol paper.**

Response: We have added the topic guides as Appendix 2 A-J and referred to them in the text.

- **As a protocol paper, it provides an opportunity to provide a summary of the fidelity framework that will be used to check that the service is being delivered as per expectations and standard. A bullet point list would be helpful outlining fidelity expectations.**

Response: Thank you for this suggestion. There are many frameworks that can be used to assess fidelity and process evaluation. In our study, we have chosen to use the Medical Research Council (MRC) process evaluation guidance as the overarching framework, which includes key implementation domains such as adoption, fidelity, reach, and dose. We selected this approach to provide a coherent and integrated framework for the overall process evaluation. We therefore do not plan to introduce an additional fidelity framework, as this would create conceptual overlap and inconsistency within the protocol. However, to address the reviewer’s request for greater clarity, we have expanded the description of fidelity to specify the aspects of intervention delivery that will be assessed, while maintaining consistency with the MRC-guided framework.

- **The set up or 'enactment' phase requires consideration with respect to dose, reach and fidelity. If the health care workers don't attend training when invited, do attend but don't engage with the training, training content is inadequate or delivery is poor then feasibility is diminished from the outset, the planned consideration of this element in the protocol would be helpful.**

Response: Thank you for this important point. We agree that feasibility may be affected from the outset by the quality and uptake of the set-up phase, particularly training of health workers. We have now clarified that we consider the training phase within our assessment of feasibility. This includes an assessment of its adoption, fidelity, reach, and dose, using training monitoring records, as well as questions relating to training experiences included in healthcare worker interviews. Interviewing trainers was something we considered at a very early stage of planning the feasibility study, however this has changed when we took the decision for training to be done by the study team. We have removed reference to trainers' interviews from the manuscript.

It is also usual, within a feasibility study protocol, to present the progression criteria that would be used to enable this to transition to a definitive trial.

Thank you for raising this important issue. We agree that it is useful for a feasibility protocol to indicate how the findings will inform a decision about progression to a future definitive trial. However, given the nature of this study of a complex intervention, we do not consider that a single numerical threshold alone would be sufficient to determine progression. Instead, progression to a definitive trial will be informed by the overall assessment of feasibility, including quantitative feasibility indicators (e.g., identification/screening, referral, treatment initiation, follow-up), together with fidelity of implementation, acceptability (among participants and health workers), and the practicality of delivery across settings. This integrated assessment will be used to determine whether the intervention and trial procedures are feasible to proceed largely as planned, feasible with modifications, or require substantial revision before a definitive trial. We have now added this clarification to the protocol under the section on 'Trial design'.

- **Finally, as a health intervention, there is no consideration of safety i.e. are the right decisions being made, are patients being unexpectedly harmed by the intervention. Some description of the monitoring for safety would be helpful. It can be part of the fidelity assessment.**

Thank you for this important comment. We agree that, even in a feasibility study, it is important to describe how participant safety and the appropriateness of clinical decision-making will be monitored. We have revised the protocol to clarify that participant safety will be monitored throughout the study through documentation of adverse effects and emerging safety concerns, prompt clinical review and

referral/escalation where indicated, as well as assessment of whether key clinical decisions are made in accordance with the protocol, with discussion of any deviations with potential safety implications during routine study management meetings. We have also clarified that these checks will be incorporated within the broader assessment of implementation fidelity and ongoing internal study oversight. The protocol now includes explicit wording in the Trial design, Outcomes/process evaluation (fidelity), Data Monitoring, and Risks sections.
